# Ocular Adverse Events Associated With Antidepressants: A Large‐Scale Data Analysis From the FAERS Database

**DOI:** 10.1002/cns.70994

**Published:** 2026-06-17

**Authors:** Yiming Peng, Yichen Cao, Hanhan Liu, Junlong Ma, Guoping Yang

**Affiliations:** ^1^ Center of Clinical Pharmacology, Third Xiangya Hospital Central South University Changsha Hunan China; ^2^ Department of Ophthalmology, Third Xiangya Hospital Central South University Changsha Hunan China; ^3^ Department of Pharmacy, National Clinical Research Center for Geriatric Diseases, Xiangya Hospital Central South University Changsha Hunan China; ^4^ Xiangya School of Pharmaceutical Sciences Central South University Changsha Hunan China

**Keywords:** antidepressants, FAERS, NDDIs, ocular adverse events, pharmacovigilance, SSRIs

## Abstract

**Objective:**

Global antidepressant use has risen substantially, raising concerns about their potential ocular adverse effects. Nevertheless, large‐scale comprehensive assessments of ocular side effects associated with various classes of antidepressants remain limited.

**Methods:**

A retrospective pharmacovigilance analysis was performed using the FDA Adverse Event Reporting System (FAERS) from Q1 2015 to Q4 2024. Reports associated with antidepressants were systematically extracted, and disproportionality analyses were performed to identify potential associations with ocular adverse events (AEs).

**Results:**

62,020 ocular AE reports were identified for 13,348 patients (median age, 44 years), with higher susceptibility observed in females. Norepinephrine–dopamine disinhibitors (NDDIs) showed the strongest association with eye disorders (IC 3.84, 95% CI 3.75–3.90), while selective serotonin reuptake inhibitors (SSRIs) showed the greatest number of positive associations across key ocular AEs. Analysis of the 36 antidepressants with sufficient case reports for ocular neuromuscular disorders revealed that 33 were associated with such disorders, most notably mydriasis, miosis, and anisocoria.

**Conclusion:**

This study delineates the ocular risks of antidepressants, highlighting higher toxicity for NDDIs and SSRIs and widespread associations with ocular neuromuscular disorders. Vigilant monitoring and early intervention are warranted to ensure safe clinical use.

## Graphical abstract

This decadal FAERS study (2015–2024) identifies 62,020 ocular adverse events (AEs) across 39 antidepressants, highlighting ocular neuromuscular disorders as a key risk. NDDIs show the strongest associations, while SSRIs exhibit the broadest signal spectrum. These findings underscore the clinical necessity for risk‐stratified, targeted clinical vigilance to mitigate treatment‐emergent visual complications.

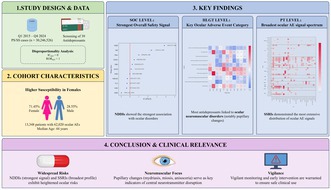

## 1 Introduction

Depression is a multifaceted psychiatric disorder that profoundly impairs social functioning and quality of life, presenting with heterogeneous clinical manifestations and imposing substantial societal burdens. In 2022, the World Health Organization ranked depressive disorders as the second leading contributor to global years lived with disability [1], affecting more than 300 million individuals worldwide [2]. Pharmacologically, selective serotonin reuptake inhibitors (SSRIs) constitute the cornerstone of treatment, while other therapeutic classes include serotonin–norepinephrine reuptake inhibitors (SNRIs), noradrenergic and specific serotonergic antidepressants (NaSSAs), norepinephrine‐dopamine reuptake inhibitors (NDRIs), norepinephrine reuptake inhibitors (NRIs), norepinephrine‐dopamine disinhibitors (NDDIs), serotonin antagonist and reuptake inhibitors (SARIs), tricyclic antidepressants (TCAs), monoamine oxidase inhibitors (MAOIs), and N‐methyl‐D‐aspartate receptor (NMDAR) antagonists [3, 4, 5]. In recent decades, antidepressant use has risen sharply [6, 7], driven both by the expansion of clinical indications beyond major depressive disorder to include anxiety disorders, chronic pain, and other conditions [8, 9], and by increasing prescription rates among non‐psychiatric physicians [10]. Within this evolving landscape, the growing use of antidepressants highlights an urgent need for a systematic evaluation of their potential safety risks.

Among these, ocular adverse events (AEs) associated with antidepressant use have emerged as a critical yet frequently under‐recognized safety concern. Given the fundamental role of visual function in daily activities, even mild ocular disturbances may result in disproportionate functional impairment, reduced independence, and diminished treatment adherence [11, 12]. Through the modulation of monoaminergic, cholinergic, and glutamatergic pathways, antidepressants may induce a broad spectrum of ophthalmic complications [13, 14, 15], ranging from acute, vision‐threatening conditions to chronic, quality‐of‐life–limiting manifestations. For instance, drug‐induced acute angle‐closure glaucoma (AACG) constitutes a medical emergency that may cause rapid and irreversible vision loss [16]. Conversely, more common manifestations, such as dry eye syndrome, often persist as chronic stressors that adversely affect patient well‐being [17]. Despite their clinical relevance, these symptoms remain under‐recognized in psychiatric practice, while ophthalmic findings are frequently not attributed to antidepressant exposure. This interdisciplinary fragmentation may lead to delayed intervention and suboptimal clinical management. Consequently, there is a pressing need for a systematic and comprehensive evaluation of the ocular safety profiles across diverse antidepressant classes and individual agents.

Prior literature has reported associations between SSRIs and an increased risk of AACG [18], while other studies have suggested potential links between SSRI and SNRI use and dry eye syndrome [19]. Additional evidence indicates that TCAs may induce mydriasis and cycloplegia [20]. Despite these observations, the extant evidence remains fragmented and is largely confined to studies examining individual drug classes or specific ocular conditions. This lack of systematic evaluation across the full spectrum of modern antidepressants precludes a comprehensive understanding of their relative safety signals. Addressing this gap requires large‐scale investigations capable of characterizing the ocular safety profiles of antidepressants at both the therapeutic class and individual drug levels. Identifying both class‐level patterns and drug‐specific risk signatures is imperative for informed clinical decision‐making, particularly for patients with pre‐existing ophthalmic vulnerabilities. In this context, pharmacovigilance analyses based on large spontaneous reporting systems provide a valuable approach for detecting potential safety signals in real‐world settings. The FDA Adverse Event Reporting System (FAERS), comprising more than 26 million reports, serves as a cornerstone of modern pharmacovigilance and offers a unique opportunity for real‐world characterization of drug‐associated toxicities [21, 22, 23]. Within this context, the present study undertakes a retrospective pharmacovigilance analysis to comprehensively evaluate the ocular safety profiles of antidepressants, aiming to provide evidence‐based insights for clinical risk management.

## 2 Materials and Methods

### 2.1 Data Sources

Quarterly datasets spanning Q1 2015 to Q4 2024 were retrieved from the FAERS (https://fis.fda.gov/extensions/FPD‐QDE‐FAERS/FPD‐QDE‐FAERS.html). Each quarterly release comprises eight component files: DEMO (patient demographics and administrative details), DRUG (drug information), REAC (adverse event codes), OUTC (patient outcomes), RPPC (report sources), THER (therapy start and end dates), and INDI (indications for use). These datasets were cross‐linked using the unique identifiers “PRIMARYID” and “CASEID”.

### 2.2 Procedures

Guided by multiple clinical guidelines [3, 24, 25] and considering the heterogeneous, mechanistically complex pharmacology of antidepressants, we selected 39 agents, including both established therapies and emerging novel compounds. The classification of these agents is detailed in Table S1. For analysis, the 39 antidepressants were grouped into 11 pharmacological classes, and reports of AEs related to eye disorders were retrieved using both generic and proprietary drug names.

**FIGURE 1:**
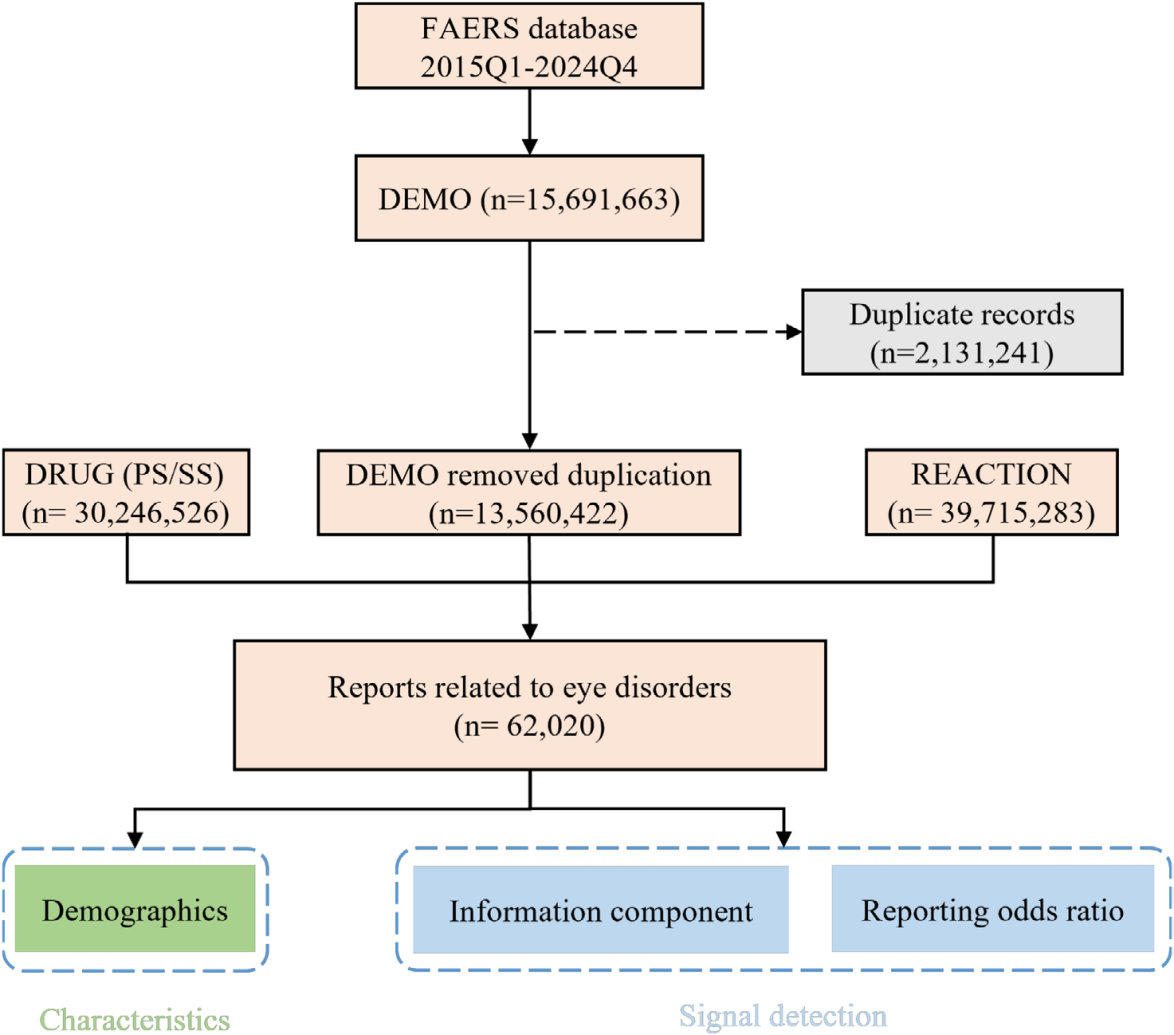
The flow chart of the study. DRUG, Drug information; DEMO, Demographic and administrative information; REACTION, Coded AEs.

The overall workflow for data processing and analysis is presented in Figure 1. Data cleaning followed the FDA‐recommended deduplication protocol: (1) The DEMO table was sorted by PRIMARYID, CASEID, and FDA_DT; among records with the same CASEID, only the most recent FDA_DT was retained. Where FDA_DT was identical, the entry with the highest PRIMARYID was selected. (2) Cases listed in the FDA deleted case files were removed from the DEMO table. (3) Records associated with deleted CASEID values were systematically excluded from all datasets.

AEs were classified according to the Medical Dictionary for Regulatory Activities (MedDRA, version 27.1) at the levels of System Organ Class (SOC), High Level Group Term (HLGT), and Preferred Term (PT). Reports were categorized as primary suspect (PS), secondary suspect (SS), concomitant (C), or interaction (I). To improve precision, analyses were restricted to reports where the antidepressant was listed as PS or SS.

### 2.3 Statistical Analysis

Disproportionality analysis, the standard approach in pharmacovigilance, was conducted using both the reporting odds ratio (ROR) and the information component (IC). A signal was considered significant when all criteria were met: IC_025_ > 0, ROR_025_ > 1, and a minimum of three reports. The mathematical definitions and parameters for these methods are provided in Table S2. To evaluate the potential influence of demographic confounding and the robustness of the detected signals, subgroup analyses stratified by sex and age (≤ 18, (18–45], (45–65], and > 65 years) were further performed for the primary ocular categories identified in the study. Data extraction was performed using SQLiteStudio (version 3.3.3), while statistical analyses were conducted with SPSS (version 27.0) and Python (version 3.10).

## 3 Results

### 3.1 Clinical Characteristics

Between Q1 2015 and Q4 2024, ocular AEs were reported in 13,348 patients. Overall, reports were disproportionately derived from female patients (*n* = 8295; 71.45%) relative to males (*n* = 3315; 28.55%). Most cases were reported from the United States (*n* = 3013; 22.57%), followed by the United Kingdom (*n* = 2755; 20.64%), Canada (*n* = 1982; 14.85%), France (*n* = 1322; 9.90%), and Germany (*n* = 615; 4.61%). The median age of the cohort was 44 years (interquartile range [IQR], 29–60), with the majority concentrated in the 18–45 years age group (*n* = 4182; 42%). With respect to onset, 3534 valid records were identified, yielding a median time to event of 0.5 days (IQR, 0.5–20.5). Notably, the majority of events occurred within 30 days of treatment initiation (*n* = 2765; 78.24%). Regarding clinical outcomes, aside from “Other outcomes” (*n* = 6409; 48.01%), hospitalization was the most commonly reported (*n* = 3658; 27.40%), followed by disability (*n* = 1289; 9.66%). Detailed demographic and clinical characteristics are summarized in Table 1.

**TABLE 1:** Demographic characteristics of patients with antidepressant‐associated ocular AEs.

Factors	Number of events (%)
*Gender*	
Data available	11,610
Female	8295 (71.45)
Male	3315 (28.55)
*Age*	
Data available	9957
≤ 18	967 (9.71)
(18–45]	4182 (42.00)
(45–65]	3040 (30.53)
(65–75]	1104 (11.09)
> 75	664 (6.67)
Median (IQR)	44.0 (29.0–60.0)
*Reported countries (Top 5)*	
United States	3013 (22.57)
United Kingdom	2755 (20.64)
Canada	1982 (14.85)
France	1322 (9.90)
Germany	615 (4.61)
*Outcomes*	
Died	626 (4.69)
Life threating	1200 (8.99)
Disabled	1289 (9.66)
Congenital Anomaly	137 (1.03)
Required intervention to prevent permanent impairment/damage	29 (0.22)
Hospitalized	3658 (27.40)
Other outcomes	6409 (48.01)
*Onset Time (d)*	
Data available	3534
≤ 30	2765 (78.24)
(30,60]	266 (7.53)
(60,90]	87 (2.46)
(90,120]	109 (3.08)
(120,150]	57 (1.61)
(150,180]	45 (1.27)
(180,360]	205 (5.80)
Median (IQR)	0.5 (0.5–20.5)

### 3.2 Overall Distribution Profile of Ocular AEs

From Q1 2015 to Q4 2024, a total of 62,020 ocular AEs associated with antidepressant use were identified in the FAERS database (Figure S1). Among these antidepressant‐related reports, venlafaxine (SNRI) contributed the largest share (12.51%), followed by citalopram (SSRI, 11.90%), sertraline (SSRI, 11.00%), and duloxetine (SNRI, 10.61%). To account for variations in prescribing patterns and market availability, we further calculated the proportion of ocular AEs within all AE reports for each agent. Agomelatine (NDDI) exhibited the highest relative burden, with 1358 ocular AEs among 12,532 total reports (10.84%), followed by maprotiline (NRI, 6.83%), opipramol (Other, 3.43%), vortioxetine (Other, 3.42%), and escitalopram (SSRI, 2.83%). Detailed distributions are provided in Table S3.

### 3.3 Association Between Antidepressants and Eye Disorders at the SOC Level

**FIGURE 2:**
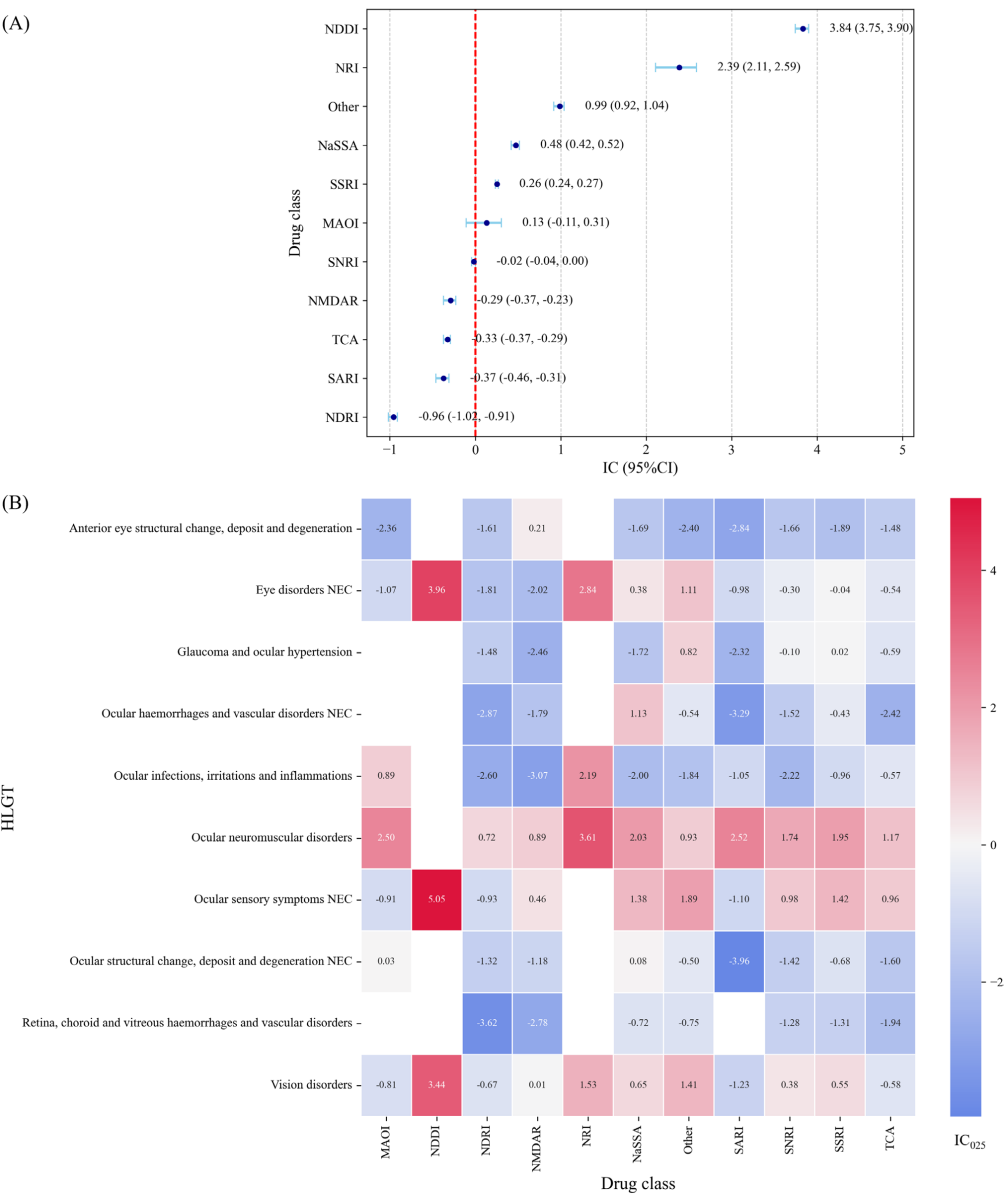
Associations between antidepressants and ocular adverse events: (A) forest plot at the System Organ Class, SOC; (B) heatmap at the High Level Group Term, HLGT. 95% CI, 95% confidence interval; MAOI, Monoamine oxidase inhibitor; NaSSA, Noradrenergic and specific serotonergic antidepressant; NDDI, norepinephrine–dopamine disinhibitor; NDRI, Norepinephrine–dopamine reuptake inhibitor; NMDAR, N‐Methyl‐D‐aspartate receptor antagonist; NRI, Norepinephrine reuptake inhibitor; SARI, Serotonin antagonist and reuptake inhibitor; SNRI, Serotonin–norepinephrine reuptake inhibitor; SSRI, Selective serotonin reuptake inhibitor; TCA, Tricyclic antidepressant.

At the SOC level of eye disorders, we systematically assessed associations across antidepressant classes. As shown in Figure 2a, when all agents were consolidated into 11 pharmacological classes, NDDIs exhibited the strongest disproportionality signal (*n* = 1358; IC_025_ = 3.75; ROR_025_ = 13.49). Signals were also detected for NRIs (*n* = 142; IC_025_ = 2.11; ROR_025_ = 4.42), Others (*n* = 2246; IC_025_ = 0.92; ROR_025_ = 1.90), NaSSAs (Reports = 3497; IC_025_ = 0.42; ROR_025_ = 1.34), and SSRIs (*n* = 27,479; IC_025_ = 0.24; ROR_025_ = 1.18). By contrast, no disproportionality signals were observed for MAOIs, SNRIs, NMDAR antagonists, TCAs, SARIs or NDRIs at the SOC level. The detailed results are presented in Table S4.

### 3.4 Association Between Antidepressant Classes and Eye Disorders at the HLGT Level

We expanded the analysis to encompass ten HLGTs, with the principal results depicted in Figure 2b. Across antidepressant classes, significant associations were concentrated within four key HLGTs: Eye disorders NEC, Ocular neuromuscular disorders, Ocular sensory symptoms NEC, and Vision disorders.

NaSSAs demonstrated the broadest spectrum of associations, contributing signals in six HLGTs: Eye disorders NEC, Ocular hemorrhages and vascular disorders NEC, Ocular neuromuscular disorders, Ocular sensory symptoms NEC, Ocular structural change, deposit and degeneration NEC, and Vision disorders. NDDIs, in contrast, displayed the strongest individual signal, most notably with Ocular sensory symptoms NEC (IC_025_: 5.05). Strikingly, ocular neuromuscular disorders emerged as the most consistent signal, with associations identified in 10 of the 11 antidepressant classes. To further validate the consistency of this prominent signal across different populations, we conducted a stratified analysis by sex and age. The disproportionality signals for the antidepressants remained statistically significant and generally stable across nearly all subgroups (Figure S2). This pattern suggests that neuromuscular ocular events represent a commonly reported signal across antidepressant classes at the HLGT level.

### 3.5 Association Between Individual Antidepressants and Eye Disorders at the HLGT Level

**FIGURE 3:**
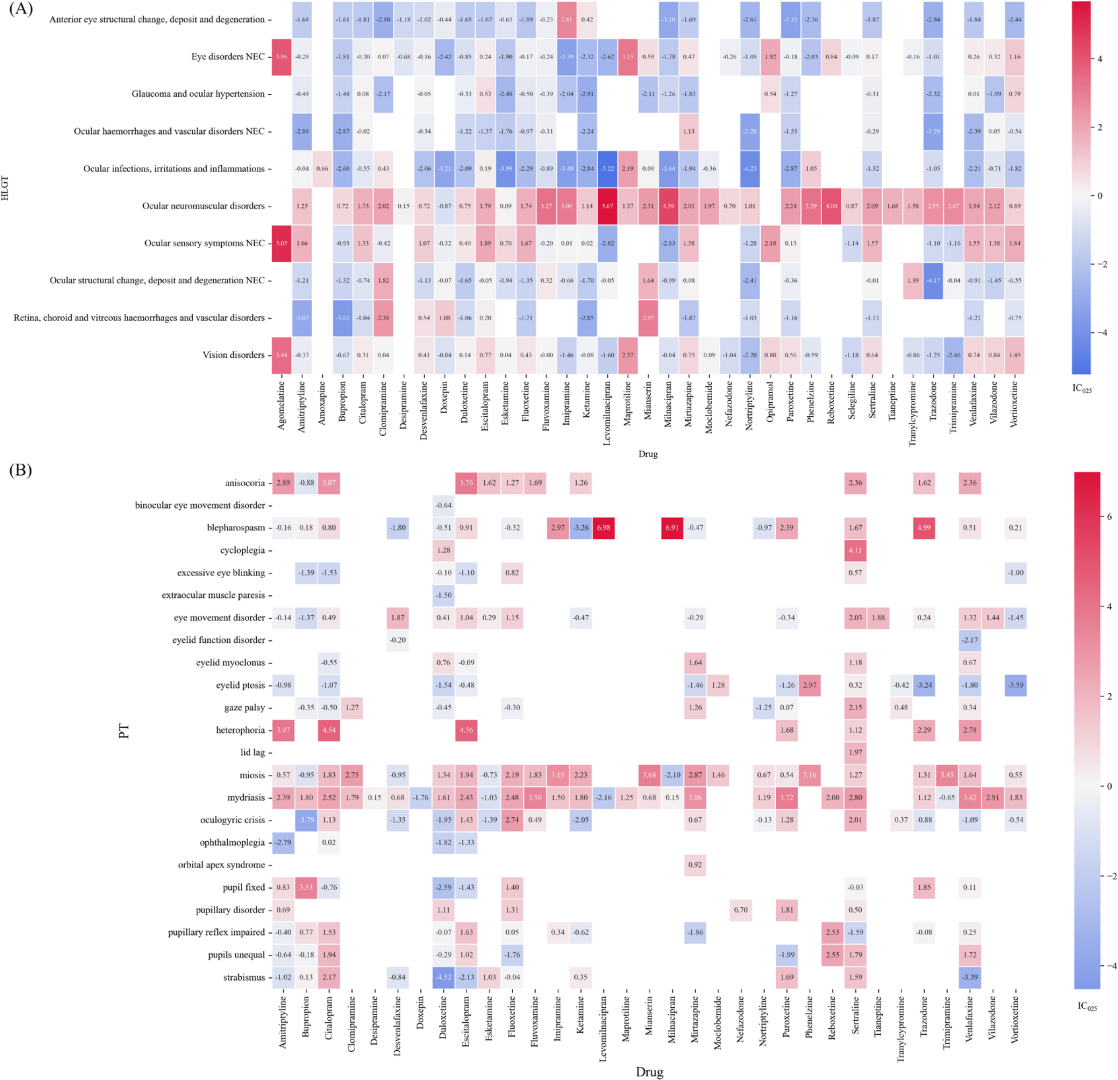
Associations between antidepressants and ocular adverse events: (A) heatmap depicting disproportionality signals across all High Group Terms (HLGTs) within the “Eye disorders” System Organ Class (SOC); (B) associations within the Ocular neuromuscular disorders HLGT at the Preferred Term (PT) level.

To provide a more granular safety profile, the 11 pharmacological classes were further disaggregated into 39 individual antidepressants, and their associations with the 10 HLGTs within the “Eye disorders” SOC were evaluated. After excluding agents with fewer than three reports, a comprehensive disproportionality map was generated for the remaining 36 compounds (Figure 3a).

The individual‐drug analysis corroborated the class‐level patterns, with significant signals predominantly concentrated in four HLGTs: Eye disorders NEC, Ocular neuromuscular disorders, Ocular sensory symptoms NEC, and Vision disorders. In contrast, other HLGTs, including Ocular infections, irritations and inflammations, and Glaucoma and ocular hypertension, showed only sporadic associations.

Among these HLGTs, Ocular neuromuscular disorders represented the most prominent signal, exhibiting the broadest drug coverage and greatest signal intensity. Notably, 33 of the 36 antidepressants (91.7%) were significantly associated with this HLGT, suggesting a widespread link between antidepressant exposure and neuromuscular ocular events. Within this category, levomilnacipran showed the strongest association (IC_025_ = 5.67). In addition, agomelatine demonstrated a distinctive multi‐domain profile, with strong signals in Ocular sensory symptoms NEC (IC_025_ = 5.05) and Eye disorders NEC (IC_025_ = 3.96). Overall, this panoramic HLGT mapping characterizes the differential distribution of ocular toxicity and identifies the predominant symptomatic clusters within the “Eye disorders” SOC.

### 3.6 Associations Between Antidepressants and Ocular Neuromuscular Disorders at the PT Level

To further delineate the relationship between antidepressants and ocular neuromuscular disorders, we extended the HLGT analysis to all subordinate PTs and evaluated their associations with individual agents (Figure 3b). Within this HLGT, most antidepressants were significantly associated with mydriasis and miosis, while anisocoria also yielded a pronounced signal. Notably, these three PTs converge on the regulation of pupillary function. Among all agents, sertraline demonstrated the most pronounced disproportionality signals at the PT level, showing robust associations across most ocular neuromuscular disorders.

### 3.7 Associations Between Antidepressants and Eye Disorders at the PT Level

We further screened all PT signals within the Eye disorders (SOC) across 39 antidepressants. Using the predefined significance threshold, 456 disproportionality signals were identified. The 30 most strongly represented PTs were then examined from two complementary perspectives: their overall reporting frequency and the number of antidepressants associated with each PT.

#### 3.7.1 Analysis of Report Frequency at the PT Level

**FIGURE 4:**
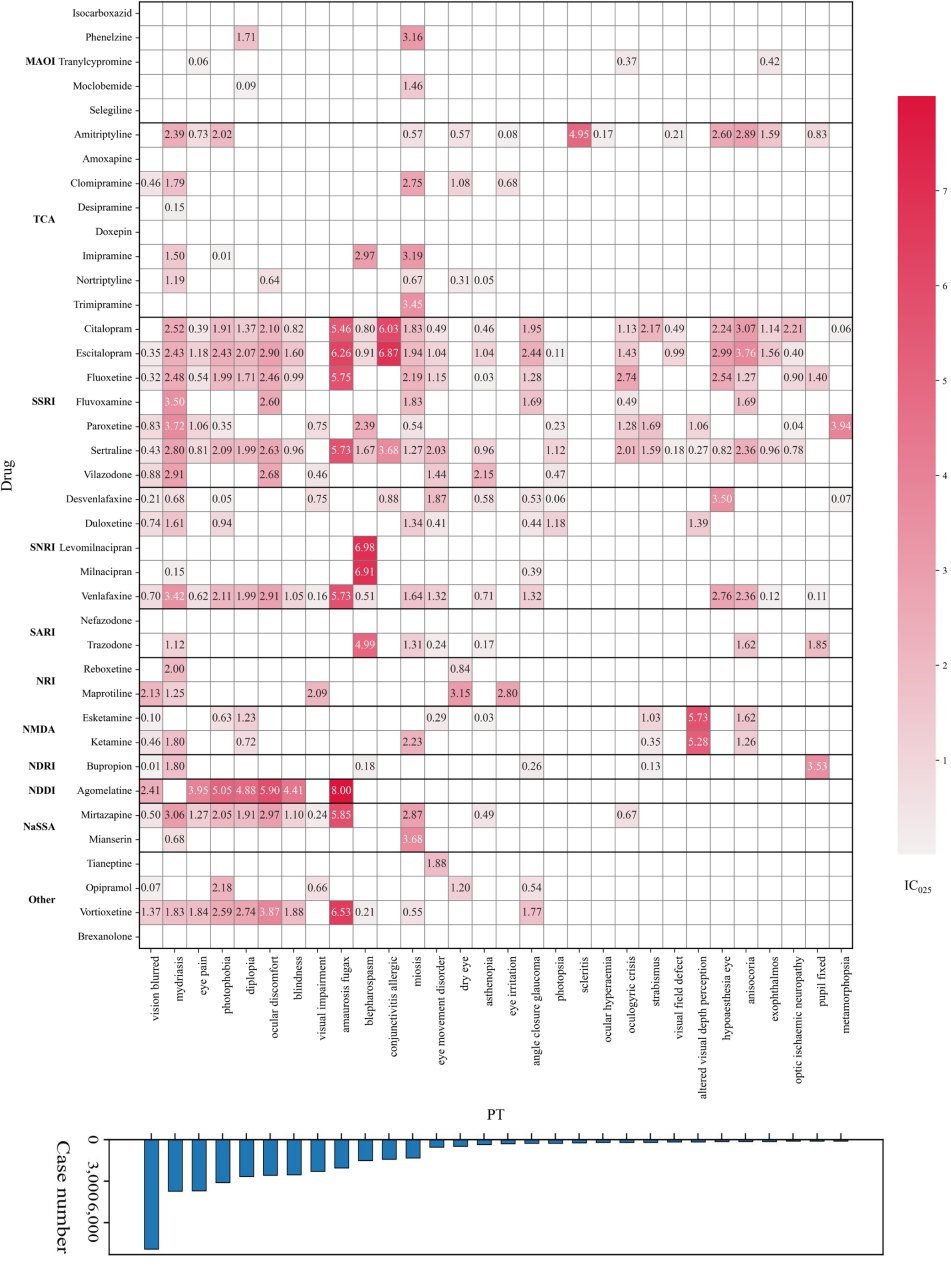
Associations between antidepressants and ocular adverse events based on report frequency. MAOI, Monoamine oxidase inhibitor; NaSSA, Noradrenergic and specific serotonergic antidepressant; PT, Preferred Term; NDDI, norepinephrine–dopamine disinhibitor; NDRI, Norepinephrine–dopamine reuptake inhibitor; NMDAR, N‐Methyl‐D‐aspartate receptor antagonist; NRI, Norepinephrine reuptake inhibitor; SARI, Serotonin antagonist and reuptake inhibitor; SNRI, Serotonin–norepinephrine reuptake inhibitor; SSRI, Selective serotonin reuptake inhibitor; TCA, Tricyclic antidepressant.

For each PT, the total number of reports was calculated, and the 30 most frequently reported PTs were subjected to detailed analysis (Figure 4). Vision blurred emerged as the most frequently reported PT (7923 cases), followed by mydriasis (3756 cases), eye pain (3727 cases), photophobia (3133 cases), and diplopia (2704 cases).

Among these 30 PTs, sertraline was associated with the greatest number of signals (22/30), followed by citalopram (21/30), escitalopram (21/30), and venlafaxine (18/30). For sertraline, the most pronounced signal was amaurosis fugax (IC_025_ = 5.73). Both citalopram and escitalopram exhibited their most pronounced association with allergic conjunctivitis (IC_025_ = 6.03 and 6.87, respectively), whereas venlafaxine also showed amaurosis fugax as its most prominent PT association (IC_025_ = 5.73).

#### 3.7.2 Analysis of the Number of Antidepressants at the PT Level

**FIGURE 5:**
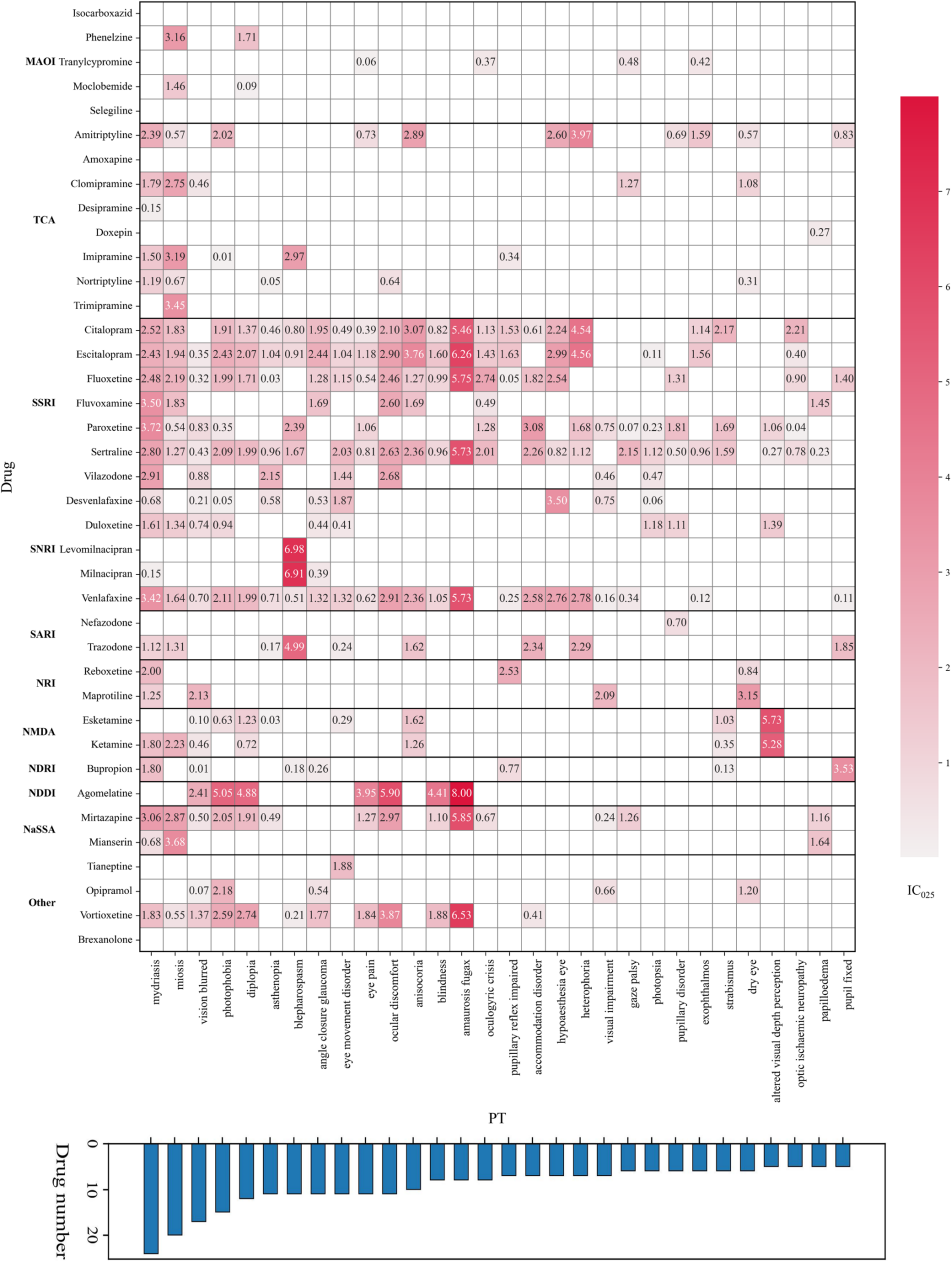
Associations between antidepressants and ocular adverse events based on the number of implicated drugs. MAOI, Monoamine oxidase inhibitor; NaSSA, Noradrenergic and specific serotonergic antidepressant; PT, Preferred Term; NDDI, norepinephrine–dopamine disinhibitor; NDRI, Norepinephrine–dopamine reuptake inhibitor; NMDAR, N‐Methyl‐D‐aspartate receptor antagonist; NRI, Norepinephrine reuptake inhibitor; SARI, Serotonin antagonist and reuptake inhibitor; SNRI, Serotonin–norepinephrine reuptake inhibitor; SSRI, Selective serotonin reuptake inhibitor; TCA, Tricyclic antidepressant.

In parallel, we quantified for each PT the number of antidepressants that generated disproportionality signals and identified the 30 PTs with the broadest antidepressant coverage for further evaluation (Figure 5). Among the 39 antidepressants assessed, mydriasis was linked to the greatest number of drugs (24/39), followed by miosis (20/39), blurred vision (17/39), photophobia (15/39), diplopia (12/39), angle‐closure glaucoma (11/39), and asthenopia (11/39).

Within this set of 30 terms, sertraline exhibited the broadest spectrum of signals (25/30), followed by venlafaxine (22/30), citalopram (21/30), escitalopram (21/30), and fluoxetine (20/30). For each of these five agents, amaurosis fugax constituted the most prominent signal.

When findings based on reporting frequency and drug number were integrated, SSRIs consistently exhibited the greatest number of ocular signals among the 30 most prominent PTs. This pattern highlights SSRIs as the class most strongly associated with core ocular adverse events, whereas other antidepressant classes demonstrated comparatively weaker signals.

Finally, evaluation of the top 30 PTs revealed several potential ocular AEs that appear underrepresented in current drug labeling or overlooked in prior studies. These findings, summarized in Table S5, encompass 32 antidepressants and include events such as amaurosis fugax, angle‐closure glaucoma, anisocoria, blepharospasm, blindness, diplopia, dry eye, eye movement disorder, ocular hypoaesthesia, miosis, mydriasis, photophobia, impaired pupillary reflex, strabismus, and visual field defect.

## 4 Discussion

Ocular AEs associated with antidepressants constitute a clinically important yet often underrecognized issue. Despite their frequent neglect in routine pharmacological practice, such reactions can markedly impair daily functioning and undermine treatment adherence, thereby raising concern within the clinical community. Previous investigations have been hampered by limited sample sizes or restricted drug‐class coverage, precluding comprehensive and reliable assessments. Leveraging a decade of FAERS data, our study encompasses a broad spectrum of antidepressants in current clinical use, providing the most comprehensive and granular post‐marketing assessment to date of antidepressant‐associated ocular risks.

### 4.1 Sex Differences

Descriptive analyses demonstrated a pronounced predominance of female reports of antidepressant‐associated ocular AEs (71.45% versus 28.55% in males), a disparity confirmed by chi‐square testing (*p* < 0.0001). This finding establishes a significant sex difference in ocular AE reporting, consistent with previous observations [26, 27]. Beyond women's higher likelihood of receiving antidepressant prescriptions, diagnoses, and treatments [28], their heightened susceptibility to ocular events may reflect underlying pharmacokinetic and pharmacodynamic distinctions [29, 30]. First, a greater proportion of body fat promotes sequestration of lipophilic antidepressants (e.g., trazodone and bupropion), prolonging their half‐life and enhancing ocular exposure. Second, metabolism through the cytochrome P450 (CYP450) system exhibits sex‐specific variability: certain isoenzymes differ in activity between men and women, and estrogen may act as a competitive substrate, together leading to reduced clearance and elevated plasma concentrations at equivalent doses. Finally, sex hormone receptors are widely expressed in ocular tissues, where estrogen and progesterone can modulate neurotransmitter systems, such as serotonin (5‐hydroxytryptamine, 5‐HT) and glutamate signaling, as well as synaptic plasticity, thereby altering drug sensitivity. Collectively, these mechanisms provide a plausible biological rationale for the observed sex differences in antidepressant‐related ocular AEs.

### 4.2 Agomelatine (NDDI) and Ocular Adverse Events

Among all antidepressant classes, agomelatine (NDDI) exhibited the most pronounced signal for ocular disorders at the SOC level, highlighting the need for careful clinical monitoring. This observation appears counterintuitive given earlier reports of ocular protection: agomelatine has been shown to lower intraocular pressure in both normotensive and hypertensive rabbit models [31], reduce intraocular pressure in patients with primary open‐angle glaucoma (POAG) [32], enhance tear secretion and potentially mitigate dry eye syndrome [33], and exert neuroprotective effects by attenuating glial inflammation and retinal cell apoptosis [34]. However, our results do not contradict these earlier observations. The detected signal was confined to seven PTs (amaurosis fugax, ocular discomfort, photophobia, diplopia, blindness, eye pain, and blurred vision), whereas no associations were observed for glaucoma, ocular hypertension, dry eye, or neuromuscular ocular disorders. Taken together, these findings suggest that agomelatine may indeed confer therapeutic benefits in specific ocular contexts, yet it is not devoid of risk. The agent exhibits a dual profile, protective in certain domains but detrimental in others, emphasizing the importance of a balanced clinical perspective and vigilant monitoring when prescribing it.

### 4.3 SSRIs and Ocular Adverse Events

In contrast to the preliminary SOC‐level analysis, SSRIs showed the most pronounced associations across the 30 PTs with the highest representation, examined from two complementary perspectives. While previous studies have reported ocular AEs with agents such as escitalopram, citalopram, sertraline, fluoxetine, and paroxetine [35], evidence regarding fluvoxamine and vilazodone has remained limited. Our analysis expands this understanding: fluvoxamine showed significant associations with mydriasis, ocular discomfort, miosis, angle‐closure glaucoma, anisocoria, papilloedema, and oculogyric crisis, whereas vilazodone exhibited signals for mydriasis, ocular discomfort, asthenopia, eye movement disorder, vision blurred, photopsia, and visual impairment. Collectively, these findings indicate that SSRIs encompass a broad and diverse spectrum of ocular risks, likely mediated by complex mechanisms. Mechanistically, SSRIs exert their effects by selectively inhibiting the presynaptic serotonin transporter, resulting in elevated synaptic 5‐HT levels [36]. Serotonin receptors are widely distributed throughout ocular tissues, including the iris–ciliary complex, cornea, and retina [37, 38], where they govern critical aspects of ocular homeostasis. Excessive serotonergic stimulation induced by SSRIs may disrupt these processes, leading to a variety of ocular AEs. Collectively, these mechanisms may underlie the particularly wide ocular risk profile observed with SSRIs. The particularly wide ocular risk profile observed for SSRIs likely reflects the pervasive role of serotonergic signaling within the eye, underscoring the need for heightened clinical vigilance in their use.

### 4.4 Antidepressants and Ocular Neuromuscular Adverse Events

A salient observation from this study is the pervasive association between antidepressant use and signals of ocular neuromuscular disorders, highlighting a potentially underrecognized safety concern. At the PT level, most antidepressants were associated with mydriasis and miosis, reflecting alterations in pupil size. Several agents were associated with both dilation and constriction, a phenomenon not previously characterized systematically. We also identified a notable signal for anisocoria, further underscoring the complex impact of antidepressants on pupillary dynamics. Clinically, pupil alterations are of particular importance owing to their ease of detection. Fluctuations in pupil diameter independent of light stimuli may reflect coordinated neuronal activity in central pupillary control pathways [39], including parasympathetic efferents from the Edinger–Westphal nucleus and sympathetic circuits originating from hypothalamic and brainstem centers [40], suggesting that the primary targets of antidepressant actions may reside in central pathways, with ocular manifestations as indirect reflections of these central effects. Our findings indicate that antidepressants exert multifaceted and heterogeneous influences on pupillary regulation. Sympathetic stimulation promotes mydriasis via contraction of the iris dilator muscle, whereas parasympathetic activity induces miosis through the iris sphincter muscle [41]. By altering serotonergic, noradrenergic, and dopaminergic transmission within the central nervous system, antidepressants may differentially modulate ocular receptors and their subtypes. For example, activation of 5‐HT1A receptors suppresses sympathetic drive to the iris dilator muscle, resulting in miosis [42, 43], whereas 5‐HT7 receptor stimulation antagonizes parasympathetic control of the sphincter muscle, producing passive mydriasis [44]. Similarly, α₁A‐adrenergic receptors mediate iris dilator contraction [45], and dopamine amplifies sympathetic norepinephrine signaling, enhancing α‐adrenergic receptor activation and thereby inducing mydriasis [46, 47]. These interacting mechanisms likely account for the heterogeneous pupillary phenotypes observed, with additional modulation by receptor selectivity, drug dosage, and interindividual genetic variation. To better understand the sources of this variability, future mechanistic studies should investigate the specific receptors and central circuits governing pupillary function, as well as the modulatory influences of drug properties and genetic differences, ultimately providing a more robust basis for risk stratification and safer clinical application.

Beyond pupillary alterations, other neuromuscular manifestations likely arise through additional mechanisms. Certain antidepressants, particularly NMDAR antagonists, may disrupt ocular motor nerve function by modulating glutamatergic transmission or blocking NMDA receptor activity [48, 49]. Widely used monoaminergic antidepressants inhibit monoamine reuptake, substantially altering synaptic neurotransmitter levels. Elevated monoamine concentrations may indirectly impair neuromuscular function, thereby affecting ocular muscles [50]. Furthermore, drugs with pronounced anticholinergic properties can provoke ciliary muscle contraction and inhibit sphincter pupillae function, thereby contributing to visual disturbances [51]. Taken together, these findings underscore the intricate neuropharmacological underpinnings of antidepressant‐associated ocular neuromuscular disorders. Enhanced monitoring of pupillary and neuromuscular changes should be prioritized both in clinical practice and during the evaluation of emerging antidepressant agents, with particular attention to early indicators of dysfunction such as pupil size alterations.

### 4.5 Limitations

While the FAERS database represents a valuable resource for pharmacovigilance, its inherent structure and reporting practices impose several unavoidable limitations. First, FAERS relies on spontaneous reporting, where data quality is often inconsistent and may be affected by underreporting, duplication, and reporting bias. Second, disproportionality analyses based on spontaneous reporting systems are primarily designed for exploratory signal detection. Therefore, the detected signals should be interpreted as potential safety signals that require further confirmation in well‐designed epidemiological or clinical studies. Third, while FAERS contains dosage information, the data are frequently incomplete or heterogeneous, which constrains robust evaluation of dose–response relationships. Despite these constraints, the present study provides meaningful insights into the ocular safety profiles of antidepressants. The breadth of antidepressants evaluated enhances the generalizability of our findings, offering clinicians practical guidance for informed prescribing and rational therapeutic decision‐making.

## 5 Conclusions

This study provides critical insights into the ocular safety risks of various antidepressants. At the level of individual agents, we identified associations between 39 antidepressants and multiple major ocular AEs, including previously under‐recognized signals not adequately captured in prescribing information or the published literature. At the class level, we conducted the first large‐scale systematic comparison of ocular risk profiles across antidepressant classes, revealing that NDDIs and SSRIs confer particularly heightened risks of ocular toxicity. Furthermore, most antidepressants exhibited associations with ocular neuromuscular disorders, in which pupillary changes were particularly pronounced, yet additional neuromuscular ocular manifestations were also observed. These findings underscore the importance of careful monitoring of such events in both clinical practice and the evaluation of emerging antidepressants. Collectively, these findings advance current understanding of antidepressant‐associated ocular toxicity and highlight the importance of early detection and timely intervention to ensure safer and more effective clinical use.

## Author Contributions

Peng Yiming: Conceptualization, Formal analysis, Methodology, Software, Data curation, Visualization, Writing – original draft. Cao Yichen: Formal analysis, Methodology, Software, Visualization. Liu Hanhan: Conceptualization, Supervision, Writing – review and editing. Ma Junlong: Project administration, Conceptualization, Methodology, Software, Supervision, Writing – review and editing. Yang Guoping: Funding acquisition, Project administration, Resources, Supervision, Software, Validation, Writing – review and editing.

## Funding

This work was supported by the National Natural Science Foundation of China (No. 82474009).

## Ethics Statement

Ethical approval was not required, as all data were publicly accessible and fully de‐identified.

## Conflicts of Interest

The authors declare no conflicts of interest.

## Supporting information


**Table S1:** Pharmacological classification of the 39 antidepressants analyzed.
**Table S2:** Equations for disproportionality analyses used in signal detection.
**Table S3:** Proportions of ocular adverse events.
**Table S4:** Detailed results of associations of antidepressant classes with eye disorders at the SOC level.
**Table S5:** Potentially underrecognized ocular adverse events associated with antidepressants.
**Figure S1:** Distribution of ocular AE reports across total AEs associated with antidepressants and within all‐cause AE reports for individual drugs.
**Figure S2:** Subgroup analysis of ocular neuromuscular disproportionality signals by age and sex.

## Data Availability

The data supporting the findings of this study were derived from the FAERS database, which is a public domain resource available without application. The raw datasets can be accessed at: https://fis.fda.gov/extensions/FPD‐QDE‐FAERS/FPD‐QDE‐FAERS.html.
