# Supplementary material for: Ocular Adverse Events Associated With Antidepressants: A Large‐Scale Data Analysis From the FAERS Database
Source: CNS Neurosci Ther. 2026 Jun 17;32(6):e70994. doi: 10.1002/cns.70994 (PMC13274231; doi:10.1002/cns.70994)
Supplement: Supplementary file 1 — Table S1: Pharmacological classification of the 39 antidepressants analyzed. Table S2: Equations for disproportionality analyses used in signal detection. Table S3: Proportions of ocular adverse events. Table S4: Detailed results of associations of antidepressant classes with eye disorders at the SOC level. Table S5: Potentially underrecognized ocular adverse events associated with antidepressants. Figure S1: Distribution of ocular AE reports across total AEs associated with antidepressants and within all‐cause AE reports for individual drugs. Figure S2: Subgroup analysis of ocular neuromuscular disproportionality signals by age and sex. [file CNS-32-e70994-s001.docx]

**Table S1.** Pharmacological classification of the 39 antidepressants analyzed.

| **Drug class** | **Drug** |
| --- | --- |
| MAOI | Isocarboxazid, Phenelzine, Tranylcypromine, Moclobemide, Selegiline |
| TCA | Amitriptyline, Amoxapine, Clomipramine, Desipramine, Doxepin, Imipramine, Nortriptyline, Trimipramine |
| SSRI | Citalopram, Escitalopram, Fluoxetine, Fluvoxamine, Paroxetine, Sertraline, Vilazodone |
| SNRI | Desvenlafaxine, Duloxetine, Levomilnacipran, Milnacipran, Venlafaxine |
| SARI | Nefazodone, Trazodone |
| NRI | Reboxetine, Maprotiline |
| NMDAR | Esketamine, Ketamine |
| NDRI | Bupropion |
| NDDI | Agomelatine |
| NaSSA | Mirtazapine, Mianserin |
| Other | Tianeptine, Opipramol, Vortioxetine, Brexanolone |

Abbreviations: MAOI, Monoamine oxidase inhibitor; TCA, Tricyclic antidepressant; SSRI, Selective serotonin reuptake inhibitor; SNRI, Serotonin–norepinephrine reuptake inhibitor; SARI, Serotonin antagonist and reuptake inhibitor; NRI, Norepinephrine reuptake inhibitor; NMDAR, N-Methyl-D-aspartate receptor antagonist; NDRI, Norepinephrine–dopamine reuptake inhibitor; NDDI, norepinephrine–dopamine disinhibitor; NaSSA, Noradrenergic and specific serotonergic antidepressant.

**Table S2.** Equations for disproportionality analyses (ROR and IC) used in signal detection.

| **Algorithm** | **Equation** | **Criteria** |
| --- | --- | --- |
| IC | N_expected_= (a+b) × (a+c)/(a+b+c+d) | IC_025_ > 0 |
|  | IC= log_2_ ((a + 0.5)/ (N_expected_ + 0.5)) |  |
|  | IC_025_= IC − 3.3 × (a + 0.5) ^−0.5^ − 2 × (a + 0.5) ^−1.5^ |  |
|  | IC_975_= IC + 2.4 × (a + 0.5) ^−0.5^ – 0.5 × (a + 0.5) ^−1.5^ |  |
| ROR | ROR= (a + 0.5)/ (N_expected_ + 0.5) | ROR_025_ > 1 |
|  | 95% CI=exp^ln (ROR) ± 1.96(1/a+1/b+1/c+1/d) ^0.5^ |  |

Abbreviations: a, number of reports containing both the target drug and target adverse event; b, number of reports containing other adverse event of the target drug; c, number of reports containing the target adverse event of other drugs; d, number of reports containing other drugs and other adverse event. 95%CI, 95% confidence interval.

**Table S3.** Proportions of ocular adverse events (AEs).

| **Drug** | **ocular AE reports** | **Proportion(A)** | **all AE reports** | **Proportion(B)** |
| --- | --- | --- | --- | --- |
| Venlafaxine | 7,761 | 12.51% | 330,330 | 2.35% |
| Citalopram | 7,382 | 11.90% | 352,910 | 2.09% |
| Sertraline | 6,822 | 11.00% | 281,214 | 2.43% |
| Duloxetine | 6,582 | 10.61% | 461,315 | 1.43% |
| Escitalopram | 5,349 | 8.62% | 188,690 | 2.83% |
| Paroxetine | 4,006 | 6.46% | 202,724 | 1.98% |
| Amitriptyline | 3,958 | 6.38% | 237,645 | 1.67% |
| Mirtazapine | 3,418 | 5.51% | 138,322 | 2.47% |
| Fluoxetine | 3,291 | 5.31% | 166,743 | 1.97% |
| Bupropion | 2,857 | 4.61% | 316,149 | 0.90% |
| Vortioxetine | 2,165 | 3.49% | 63,235 | 3.42% |
| Trazodone | 1,397 | 2.25% | 113,690 | 1.23% |
| Agomelatine | 1,358 | 2.19% | 12,532 | 10.84% |
| Ketamine | 1,035 | 1.67% | 84,462 | 1.23% |
| Desvenlafaxine | 875 | 1.41% | 46,364 | 1.89% |
| Milnacipran | 581 | 0.94% | 26,527 | 2.19% |
| Esketamine | 565 | 0.91% | 48,004 | 1.18% |
| Levomilnacipran | 383 | 0.62% | 18,541 | 2.07% |
| Nortriptyline | 374 | 0.60% | 61,741 | 0.61% |
| Clomipramine | 368 | 0.59% | 21,254 | 1.73% |
| Vilazodone | 360 | 0.58% | 13,693 | 2.63% |
| Fluvoxamine | 269 | 0.43% | 12,733 | 2.11% |
| Doxepin | 179 | 0.29% | 23,015 | 0.78% |
| Imipramine | 115 | 0.19% | 8,673 | 1.33% |
| Maprotiline | 111 | 0.18% | 1,625 | 6.83% |
| Phenelzine | 79 | 0.13% | 3,876 | 2.04% |
| Mianserin | 79 | 0.13% | 10,267 | 0.77% |
| Opipramol | 71 | 0.11% | 2,069 | 3.43% |
| Tranylcypromine | 46 | 0.07% | 4,094 | 1.12% |
| Trimipramine | 38 | 0.06% | 4,680 | 0.81% |
| Selegiline | 36 | 0.06% | 3,927 | 0.92% |
| Reboxetine | 31 | 0.05% | 1,494 | 2.07% |
| Moclobemide | 27 | 0.04% | 3,349 | 0.81% |
| Nefazodone | 19 | 0.03% | 1,646 | 1.15% |
| Desipramine | 12 | 0.02% | 1,442 | 0.83% |
| Amoxapine | 9 | 0.01% | 646 | 1.39% |
| Tianeptine | 8 | 0.01% | 1,651 | 0.48% |
| Isocarboxazid | 2 | 0.00% | 129 | 1.55% |
| Brexanolone | 2 | 0.00% | 1,014 | 0.20% |

**Note: Proportion (A)**: The specific drug’s ocular AE reports relative to all antidepressant-associated ocular AE reports (N = 62,020); **Proportion (B)**: the ocular AE reports relative to the total all-cause AE reports for that specific drug.

**Table S4.** Detailed results of associations of antidepressant classes with eye disorders at the System Organ Class (SOC) level.

| **Drug class** | **Cases** | **ROR (95% CI)** | **IC (95% CI)** |
| --- | --- | --- | --- |
| NDDI | 1358 | 14.27 (13.49, 15.10) | 3.84 (3.75, 3.90) |
| NRI | 142 | 5.23 (4.42, 6.19) | 2.39 (2.11, 2.59) |
| Other | 2246 | 1.98 (1.90, 2.07) | 0.99 (0.92, 1.04) |
| NaSSA | 3497 | 1.39 (1.34, 1.44) | 0.48 (0.42, 0.52) |
| SSRI | 27479 | 1.19 (1.18, 1.21) | 0.26 (0.24, 0.27) |
| SNRI | 16182 | 0.99 (0.97, 1.00) | -0.02 (-0.04, 0.00) |
| MAOI | 190 | 1.10 (0.95, 1.26) | 0.13 (-0.11, 0.31) |
| NMDAR | 1600 | 0.82 (0.78, 0.86) | -0.29 (-0.37, -0.23) |
| TCA | 5053 | 0.80 (0.78, 0.82) | -0.33 (-0.37, -0.29) |
| SARI | 1416 | 0.77 (0.73, 0.81) | -0.37 (-0.46, -0.31) |
| NDRI | 2857 | 0.52 (0.50, 0.53) | -0.96 (-1.02, -0.91) |

Abbreviations: MAOI, Monoamine oxidase inhibitor; TCA, Tricyclic antidepressant; SSRI, Selective serotonin reuptake inhibitor; SNRI, Serotonin–norepinephrine reuptake inhibitor; SARI, Serotonin antagonist and reuptake inhibitor; NRI, Norepinephrine reuptake inhibitor; NMDAR, N-Methyl-D-aspartate receptor antagonist; NDRI, Norepinephrine–dopamine reuptake inhibitor; NDDI, Norepinephrine–dopamine disinhibitor; NaSSA, Noradrenergic and specific serotonergic antidepressant.

**Table S5.** Potentially underrecognized ocular adverse events associated with antidepressants.

| Drug | Adverse Event |
| --- | --- |
| Agomelatine | diplopia, blindness, amaurosis fugax, photophobia |
| Amitriptyline | dry eye, visual field defect, miosis, photophobia, hypoaesthesia eye, anisocoria |
| Bupropion | pupillary reflex impaired, strabismus, blepharospasm |
| Citalopram | eye movement disorder, pupillary reflex impaired, blindness, strabismus, visual field defect, amaurosis fugax, miosis, blepharospasm, hypoaesthesia eye, anisocoria |
| Clomipramine | mydriasis, dry eye, miosis |
| Desipramine | mydriasis |
| Desvenlafaxine | eye movement disorder, photophobia, hypoaesthesia eye |
| Duloxetine | eye movement disorder, miosis, photophobia |
| Escitalopram | eye movement disorder, pupillary reflex impaired, blindness, visual field defect, amaurosis fugax, miosis, photophobia, hypoaesthesia eye, anisocoria |
| Esketamine | eye movement disorder, strabismus, anisocoria |
| Fluoxetine | eye movement disorder, pupillary reflex impaired, amaurosis fugax, miosis, hypoaesthesia eye, anisocoria |
| Fluvoxamine | miosis, anisocoria |
| Imipramine | pupillary reflex impaired, miosis, blepharospasm, photophobia |
| Ketamine | mydriasis, strabismus, miosis, anisocoria |
| Levomilnacipran | blepharospasm |
| Maprotiline | dry eye |
| Mianserin | mydriasis, miosis |
| Mirtazapine | mydriasis, blindness, amaurosis fugax, miosis, photophobia |
| Moclobemide | diplopia, miosis |
| Milnacipran | angle closure glaucoma |
| Nortriptyline | mydriasis, dry eye, miosis |
| Opipramol | dry eye, photophobia |
| Paroxetine | strabismus, miosis, blepharospasm |
| Phenelzine | diplopia, miosis |
| Reboxetine | mydriasis, dry eye, pupillary reflex impaired |
| Sertraline | eye movement disorder, strabismus, amaurosis fugax, miosis, blepharospasm, hypoaesthesia eye |
| Tianeptine | eye movement disorder |
| Trazodone | mydriasis, eye movement disorder, miosis, blepharospasm, anisocoria |
| Trimipramine | miosis |
| Venlafaxine | eye movement disorder, pupillary reflex impaired, amaurosis fugax, miosis, blepharospasm, hypoaesthesia eye, anisocoria |
| Vilazodone | mydriasis, eye movement disorder |
| Vortioxetine | mydriasis, diplopia, blindness, amaurosis fugax, angle closure glaucoma, miosis, blepharospasm, photophobia |


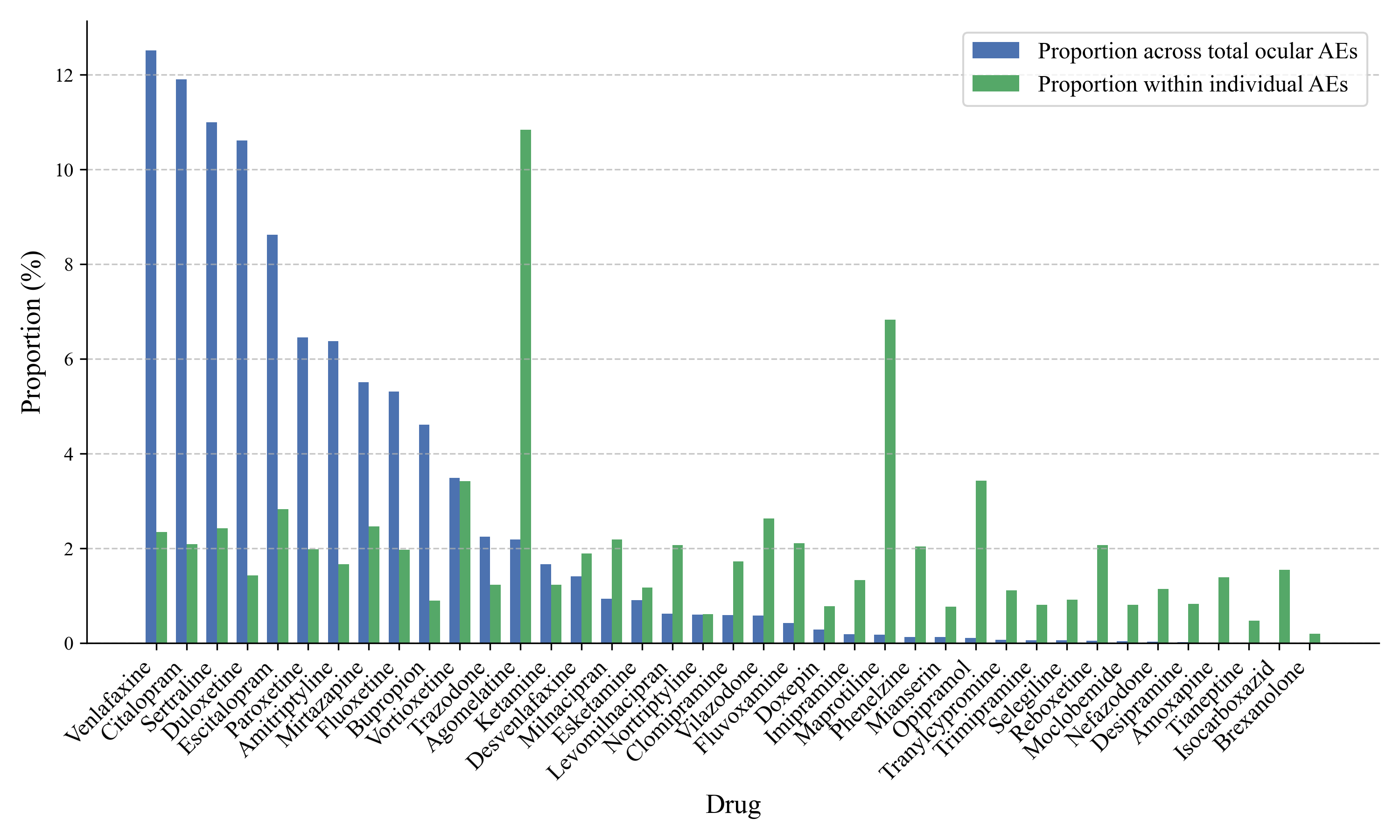


**Figure S1.** Distribution of ocular AE reports across total AEs associated with antidepressants and within all-cause AE reports for individual drugs.

**
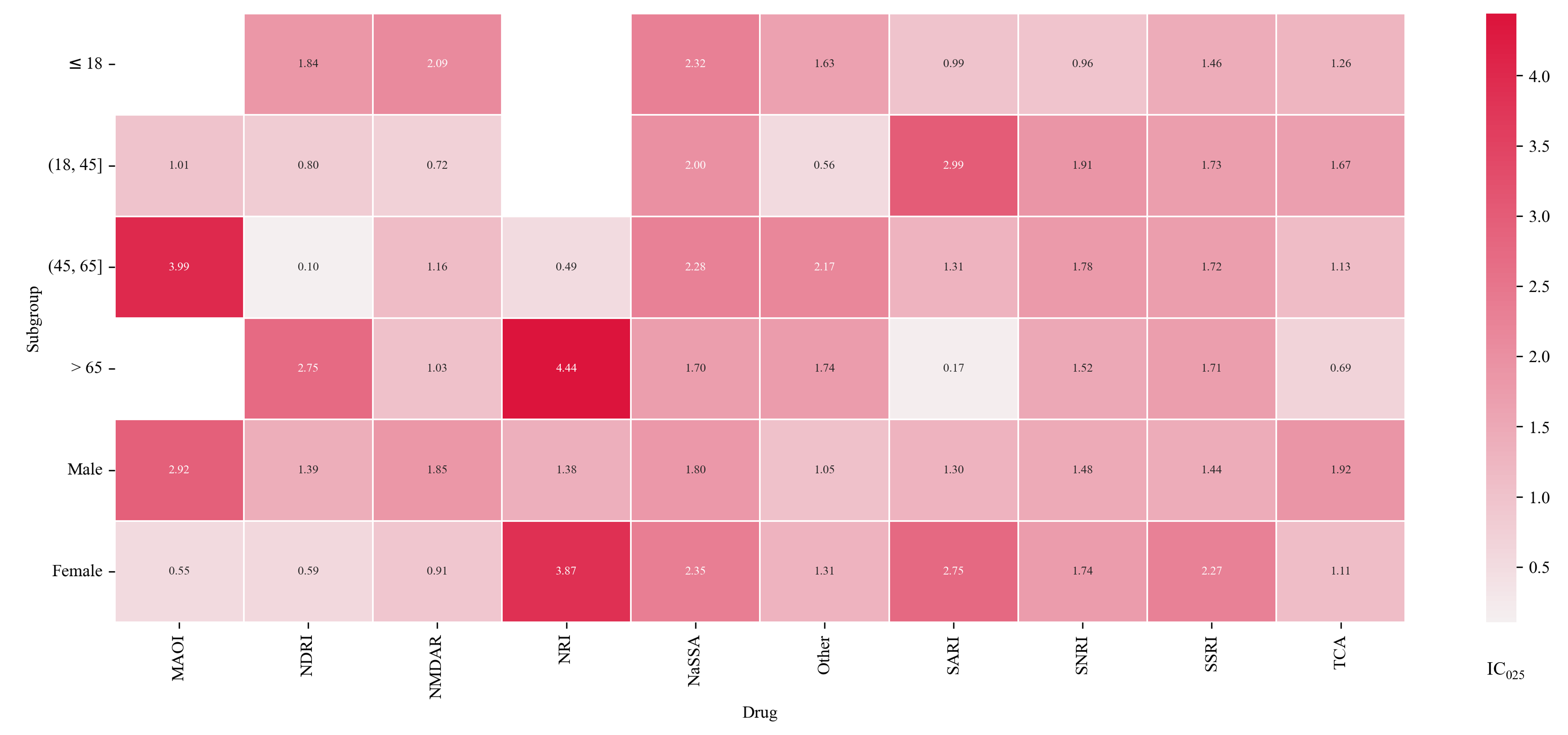
**

**Figure S2.** Subgroup analysis of ocular neuromuscular disproportionality signals by age and sex.

Age subgroups are defined as follows: ≤18 years, (18–45] years, (45–65] years, and >65 years.

Abbreviations: MAOI, Monoamine oxidase inhibitor; NDRI, Norepinephrine–dopamine reuptake inhibitor; NMDAR, N-Methyl-D-aspartate receptor antagonist; NRI, Norepinephrine reuptake inhibitor; NaSSA, Noradrenergic and specific serotonergic antidepressant; SARI, Serotonin antagonist and reuptake inhibitor; SNRI, Serotonin–norepinephrine reuptake inhibitor; SSRI, Selective serotonin reuptake inhibitor; TCA, Tricyclic antidepressant; PT, Preferred Term.
